# Supplementary material for: Disentangling Relationships among the Alpine Species of Luzula Sect. Luzula (Juncaceae) in the Eastern Alps
Source: Plants (Basel). 2023 Feb 20;12(4):973. doi: 10.3390/plants12040973 (PMC9960804; doi:10.3390/plants12040973)
Supplement: Supplementary file 1 [file plants-12-00973-s001.zip › Supple_Fig_1.pdf]

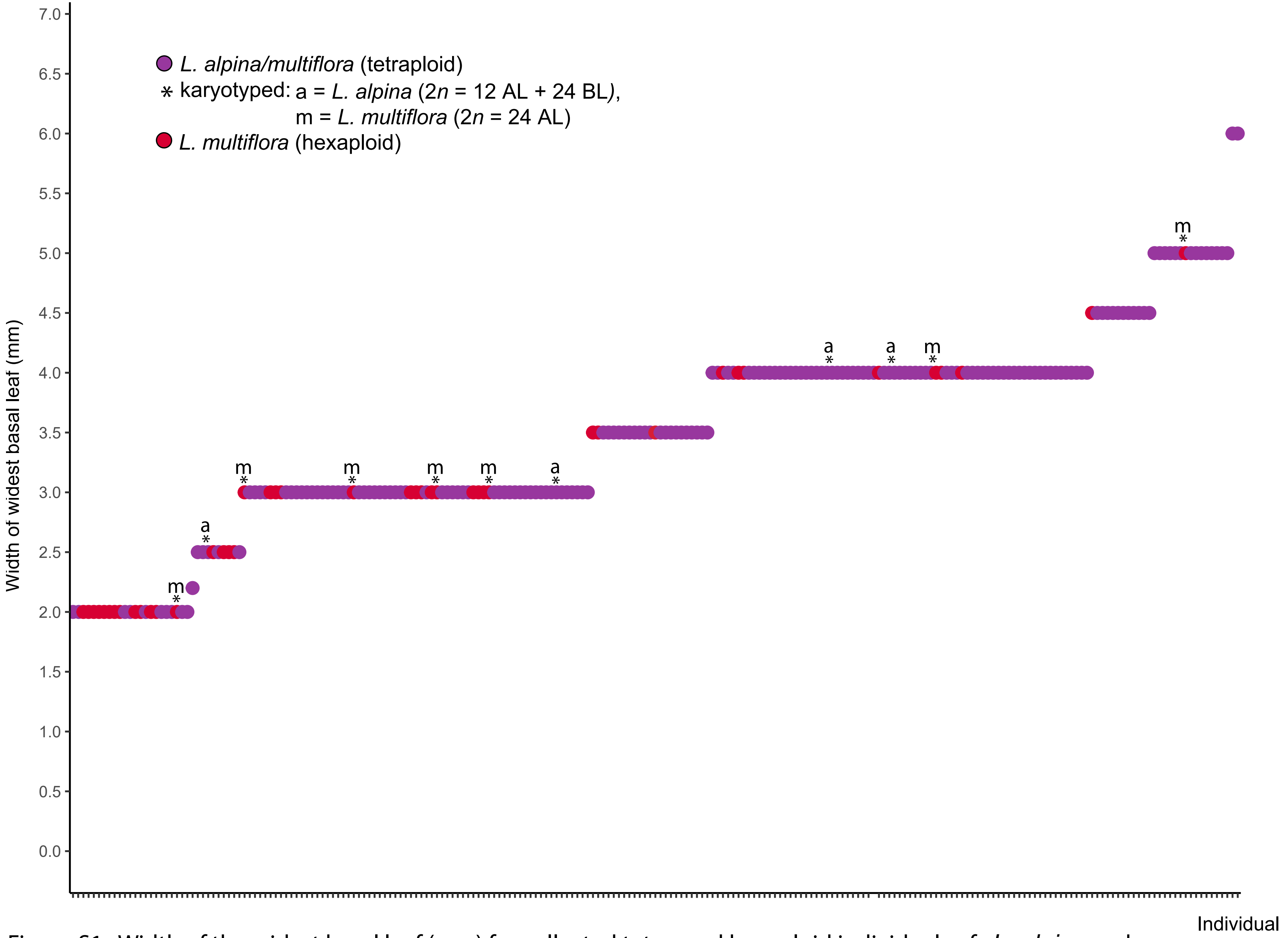

Figure S1: Width of the widest basal leaf (mm) for collected tetra- and hexaploid individuals of *L. alpina* and *L. multiflora*. Asterisks indicate karyotyped individuals labelled with the first letter of the epithet.
