# Supplementary figures and images for: Disentangling Relationships among the Alpine Species of Luzula Sect. Luzula (Juncaceae) in the Eastern Alps
Source: Plants (Basel). 2023 Feb 20;12(4):973. doi: 10.3390/plants12040973 (PMC9960804; doi:10.3390/plants12040973)

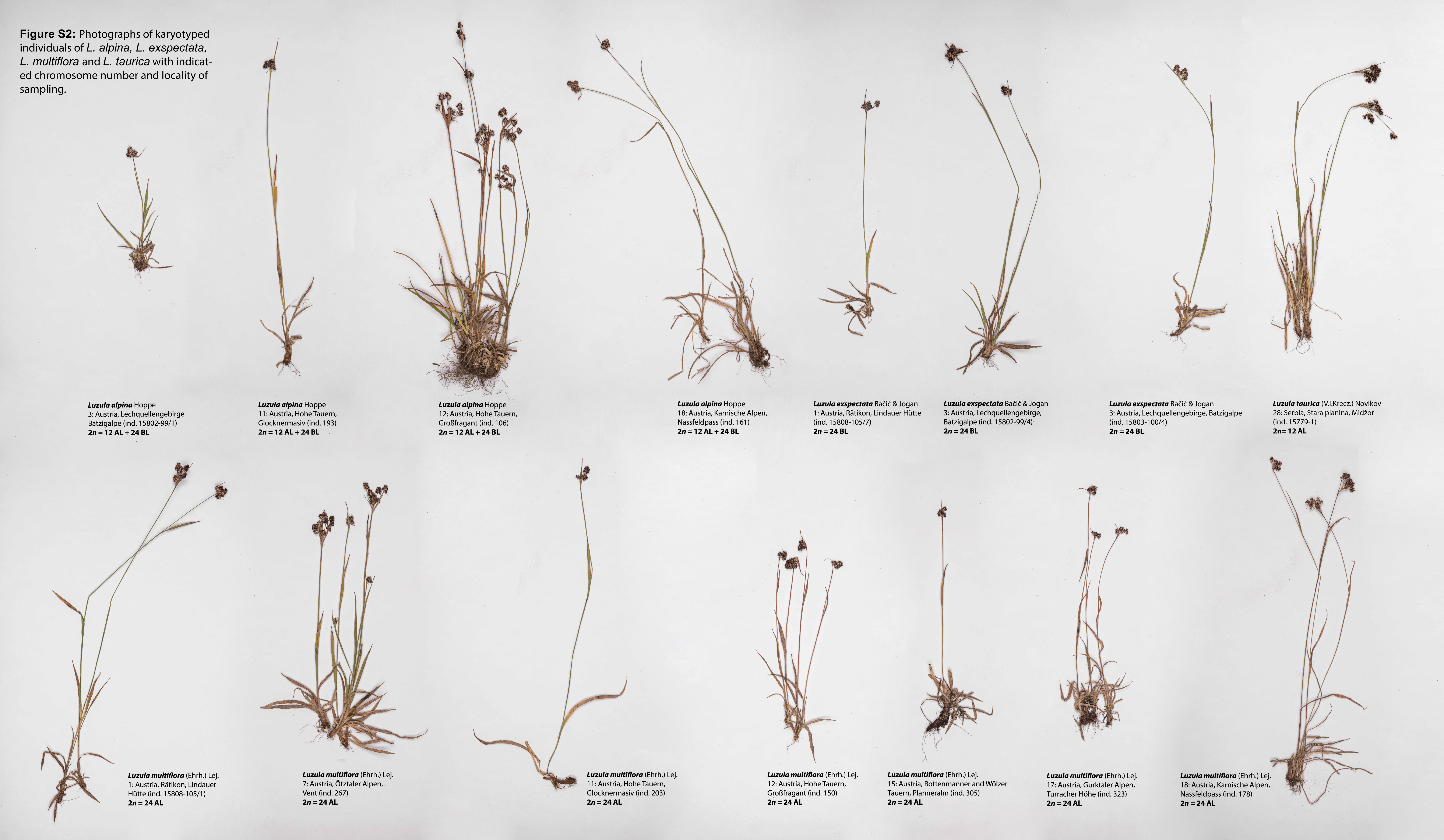

Supplement: Supplementary file 1 [file plants-12-00973-s001.zip › Suppl_Fig_2_Karyotyped.jpg]
